# Supplementary material for: Nonequivalent After-Effects of Alternating Current Stimulation on Motor Cortex Oscillation and Inhibition: Simulation and Experimental Study
Source: Brain Sci. 2022 Jan 31;12(2):195. doi: 10.3390/brainsci12020195 (PMC8870173; doi:10.3390/brainsci12020195)
Supplement: Supplementary file 1 [file brainsci-12-00195-s001.zip › brainsci-1543844-supplementary.pdf]

A. Electric Field Computational Analysis

A potential primary source of variability of transcranial electrical stimulation, including tACS, is the anatomical difference between individuals that changes the electric fields in the brain [1]. It becomes crucial guiding and optimizing the montage position at individual [2,3], or group-level [1,4]. The development of sophisticated computational models allows studying the effects of individual differences using simulations [5–8].

In our study, the head models of eighteen individuals were constructed from T1- and T2-weighted images (available on http://hdl.handle.net/1926/1687). The sample population's mean age was 43.4 ± 9.8 years (all individuals are male and neurologically healthy). The models were segmented into 14 tissues/body fluids as described in detail in [1].

The head model is treated as a passive volume conductor to compute the generated electric field produced by the injected current via the electrode montage selected. The computational method has been reported in detail in [9], and the tissue electrical conductivities have been reported previously [10]. Briefly, the electric scalar potential was determined using the scalar-potential finite-difference method [11] (0.5 mm side length) of the voxelized head model, and the electric field along the edge of the voxel was obtained by dividing the voltage between the nodes of the voxel by the distance across the nodes. The electrodes were modelled as rubber pads (0.1 S/m), identical to the size and thickness of the ones used in experiments, and placed in different positions according to the 10–20 electroencephalogram system. A layer of conductive electrode paste (thickness of 2.5 mm, conductivity of 1.6 S/m) was added between the electrodes and the scalp. The current intensity was 0.3 mA as the experiment. The frequency was assumed to have a negligible effect on the tissue conductivities for the frequencies used in this study.

The group-level analysis corresponds to the electric field in a population. The group-level electric field is obtained by averaging the electrical field on each individual's cortex projected on

the standard brain space. The procedure to register the electric field surface data on the cortex brain on the standard brain template was similar to that used previously [1]. In this work, we focus on the normal component of the electric field to the cortical surface as neuromodulation's physical quantity [12]. Finally, we obtain the mean value of the group-level in the hand-knob region [13]. Then, the inter-individual effect was quantified by the relative standard deviation [4].

References

[1] I. Laakso, S. Tanaka, S. Koyama, V. De Santis, and A. Hirata, “Inter-subject variability in electric fields of motor cortical tDCS,” *Brain Stimul.*, vol. 8, no. 5, pp. 906–913, 2015, doi: 10.1016/j.brs.2015.05.002.

[2] A. Opitz, E. Yeagle, A. Thielscher, C. Schroeder, A. D. Mehta, and M. P. Milham, “On the importance of precise electrode placement for targeted transcranial electric stimulation,” *Neuroimage*, vol. 181, pp. 560–567, Nov. 2018, doi: 10.1016/j.neuroimage.2018.07.027.

[3] F. H. Kasten, K. Duecker, M. C. Maack, A. Meiser, and C. S. Herrmann, “Integrating electric field modeling and neuroimaging to explain inter-individual variability of tACS effects,” *Nat. Commun.*, vol. 10, no. 1, pp. 1–11, Dec. 2019, doi: 10.1038/s41467-019-13417-6.

[4] J. Gomez-Tames, A. Asai, and A. Hirata, “Significant Group-Level Hotspots Found in Deep Brain Regions during tDCS: A Computational Analysis of Electric Field,” *Clin. Neurophysiol.*, vol. 3, pp. 755--765., 2020.

[5] Y. Huang, A. Datta, M. Bikson, and L. C. Parra, “Realistic volumetric-approach to simulate transcranial electric stimulation-ROAST-a fully automated open-source pipeline.,” *J. Neural Eng.*, vol. 16, no. 5, p. 56006, 2019, doi: 10.1088/1741-2552/ab208d.

[6] G. Csifcsák, N. M. Boayue, O. Puonti, A. Thielscher, and M. Mittner, “Effects of transcranial direct current stimulation for treating depression: A modeling study,” *J. Affect. Disord.*, vol. 234, pp. 164–173, 2018, doi: 10.1016/j.jad.2018.02.077.

[7] M. Parazzini, S. Fiocchi, E. Rossi, A. Paglialonga, and P. Ravazzani, “Transcranial Direct Current Stimulation: Estimation of the Electric Field and of the Current Density in an Anatomical Human Head Model,” *IEEE Trans. Biomed. Eng.*, vol. 58, no. 6, pp. 1773–1780, 2011, doi: 10.1109/TBME.2011.2116019.

[8] A. Datta, J. M. Baker, M. Bikson, and J. Fridriksson, “Individualized model predicts

brain current flow during transcranial direct-current stimulation treatment in responsive stroke patient,” *Brain Stimul.*, vol. 4, no. 3, pp. 169–174, 2011, doi: 10.1016/j.brs.2010.11.001.

[9] I. Laakso and A. Hirata, “Fast multigrid-based computation of the induced electric field for transcranial magnetic stimulation,” *Phys. Med. Biol.*, vol. 57, no. 23, pp. 7753–7765, 2012, doi: 10.1088/0031-9155/57/23/7753.

[10] I. Laakso and A. Hirata, “Computational analysis shows why transcranial alternating current stimulation induces retinal phosphenes,” *J. Neural Eng.*, vol. 10, no. 4, p. 46009, 2013, doi: 10.1088/1741-2560/10/4/046009.

[11] T. W. Dawson and M. A. Stuchly, “High-resolution organ dosimetry for human exposure to low-frequency magnetic fields,” *IEEE Trans. Magn.*, vol. 34, no. 3, pp. 708–718, 1998, doi: 10.1109/20.668071.

[12] I. Laakso, M. Mikkonen, S. Koyama, A. Hirata, and S. Tanaka, “Can electric fields explain inter-individual variability in transcranial direct current stimulation of the motor cortex?,” *Sci. Rep.*, vol. 9, no. 1, p. 626, 2019, doi: 10.1038/s41598-018-37226-x.

[13] T. A. Yousry *et al.*, “Localization of the motor hand area to a knob on the precentral gyrus. A new landmark,” *Brain*, pp. 141–157, 1997, [Online]. Available: http://www.ncbi.nlm.nih.gov/pubmed/9055804.
